# Supplementary material for: Anthraquinone-Polyaniline-Integrated Textile Platforms for In Situ Electrochemical Production of Hydrogen Peroxide for Microbial Deactivation
Source: Polymers (Basel). 2023 Jun 28;15(13):2859. doi: 10.3390/polym15132859 (PMC10361206; doi:10.3390/polym15132859)
Supplement: Supplementary file 1 [file polymers-15-02859-s001.zip › polymers-2452404-supplementary.pdf]

Article

Anthraquinone-Polyaniline-Integrated Textile Platforms for
In Situ Electrochemical Production of Hydrogen Peroxide for Microbial Deactivation

Samuel M. Mugo *, Weihao Lu and Scott Robertson

Physical Sciences Department, MacEwan University, 10700-104 Avenue, Edmonton, AB T5J 4S2, Canada; luw6@mymacewan.ca (W.L.); robertsons57@mymacewan.ca (S.R.)

* Correspondence: mugos@macewan.ca; Tel.: +1-780-633-3493; Fax: +1-780-497-5655

Supporting Information

a) CNT/CNC@textile patch

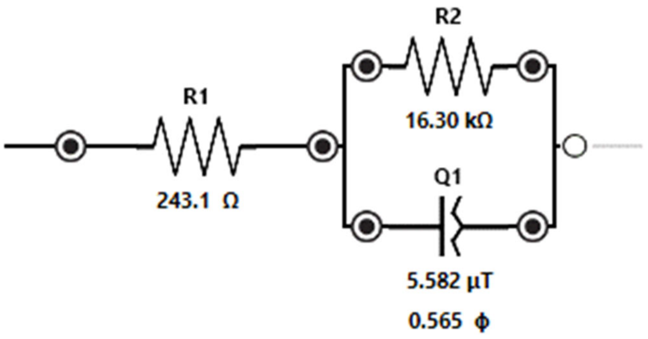

b) PANI@CNT/CNC@textile patch

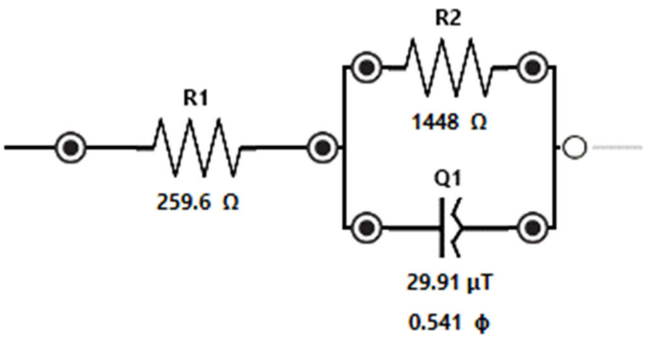

c) AQ@CNT/CNC@textile patch

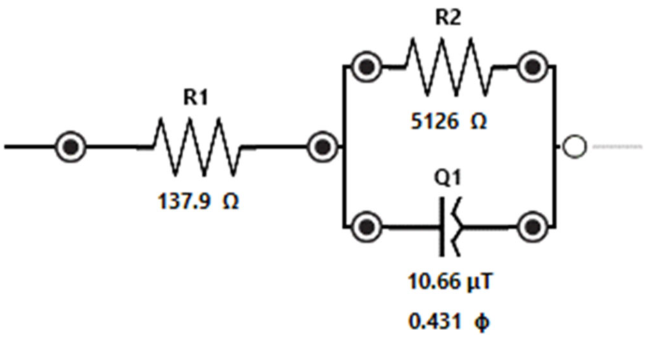

d) AQ@PANI@CNT/CNC@textile antibacterial patch

Figure S1. EIS circuit fitting for different variations of the fabricated patches.

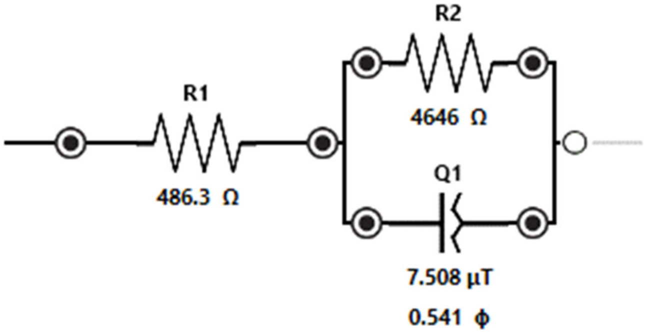

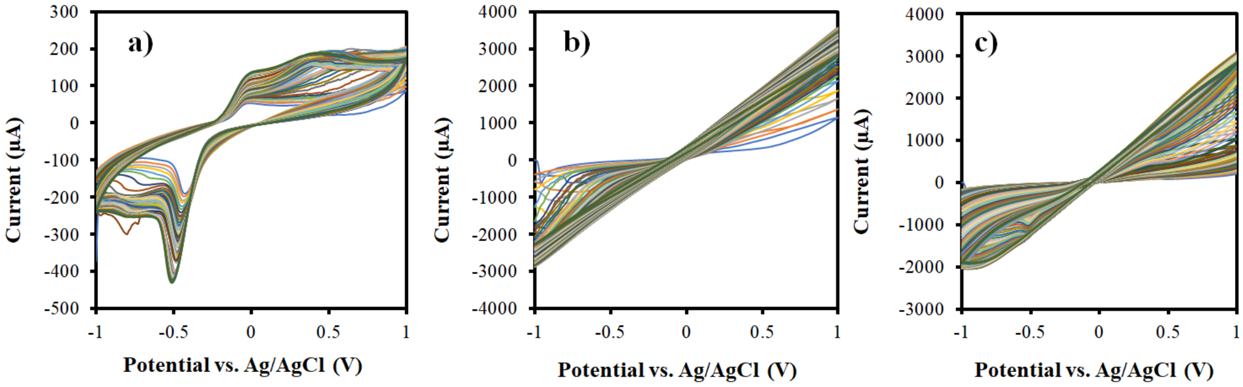

Figure S2. Overlaid CVs taken using **a)** CNT/CNC@textile; **b)** PANI@CNT/CNC@textile; and **c)** AQ@PANI@CNT/CNC@textile patches to stimulate the electrochemical production of H_2O_2.

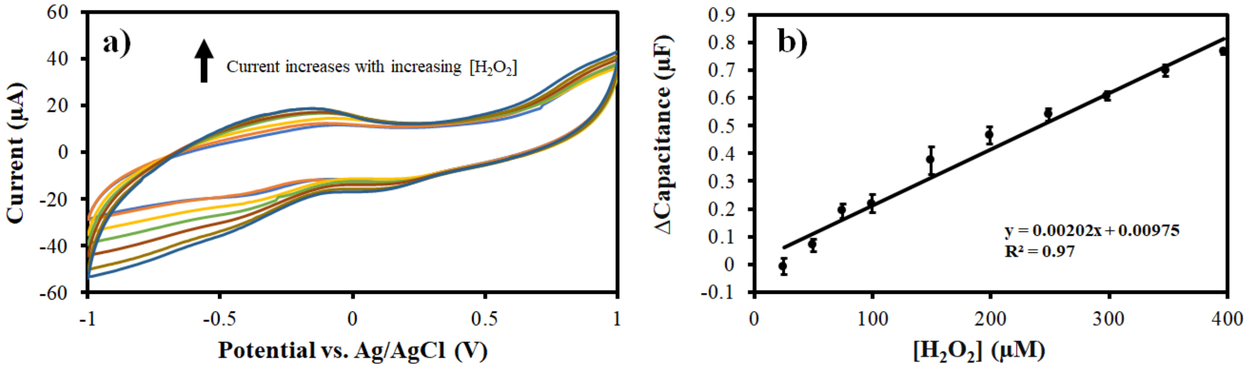

Figure S3. a) Overlaid CVs taken using the CNT/CNC sensor electrode in response to increasing H_2O_2 concentrations and **b)** resulting Δcapacitance vs. H_2O_2 concentration calibration curve.

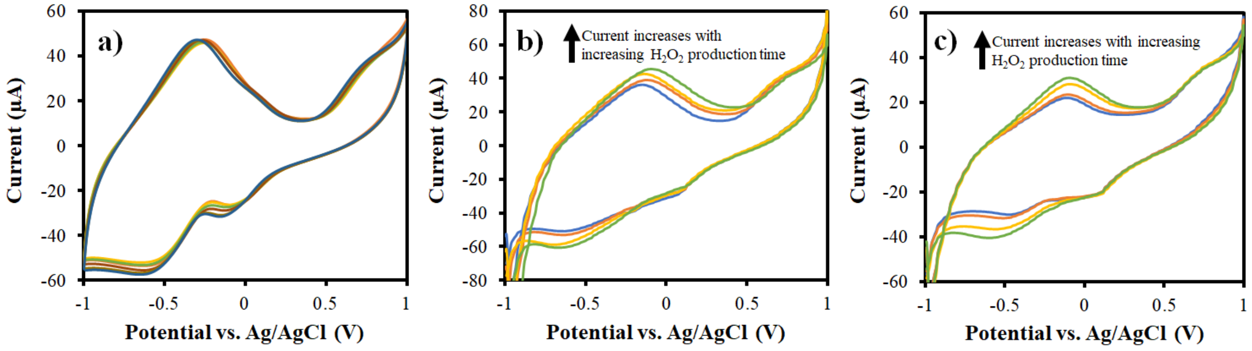

Figure S4. CVs taken using the CNT/CNC sensor electrode for the electrochemical detection of H_2O_2 produced from **a)** CNT/CNC@textile; **b)** PANI@CNT/CNC@textile; and **c)** AQ@PANI@CNT/CNC@textile patches.

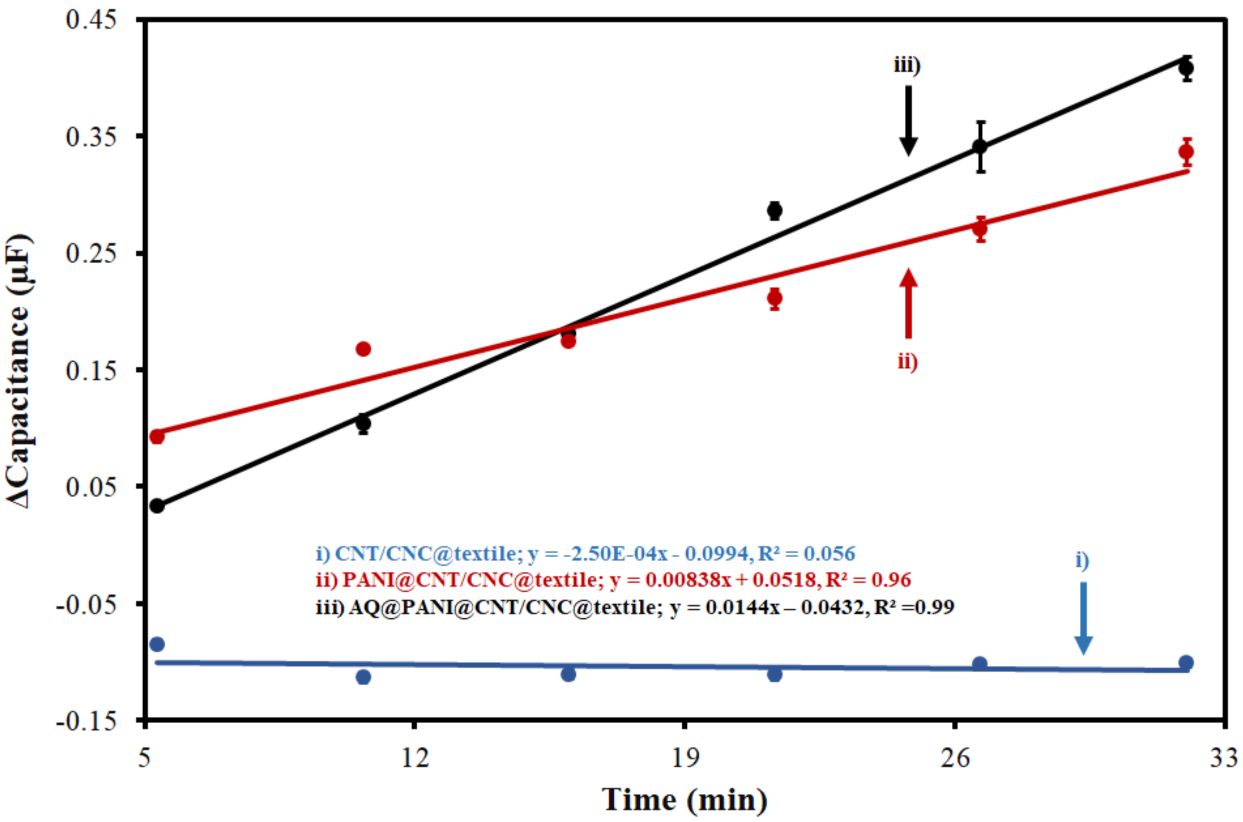

Figure S5. ΔCapacitance vs. H_2O_2 generation time plot, and resulting linear equations for **i)** CNT/CNC@textile; **ii)** PANI@CNT/CNC@textile; and **iii)** AQ@PANI@CNT/CNC@textile patches.

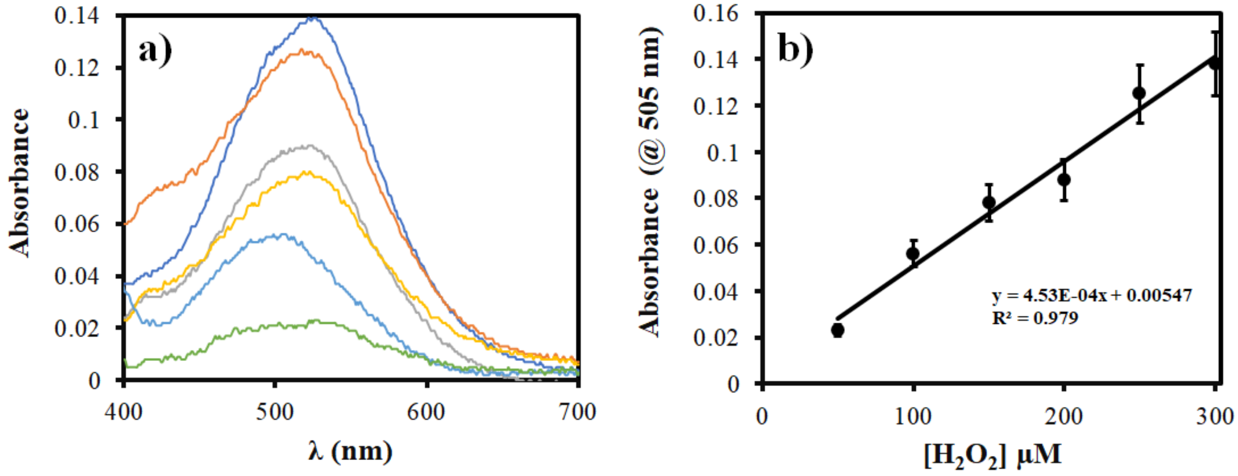

Figure S6. a) Absorption spectra for the colorimetric detection of H_2O_2 in response to increasing H_2O_2 concentrations and **b)** resulting absorbance (@ 505 nm) vs. H_2O_2 concentration calibration curve.

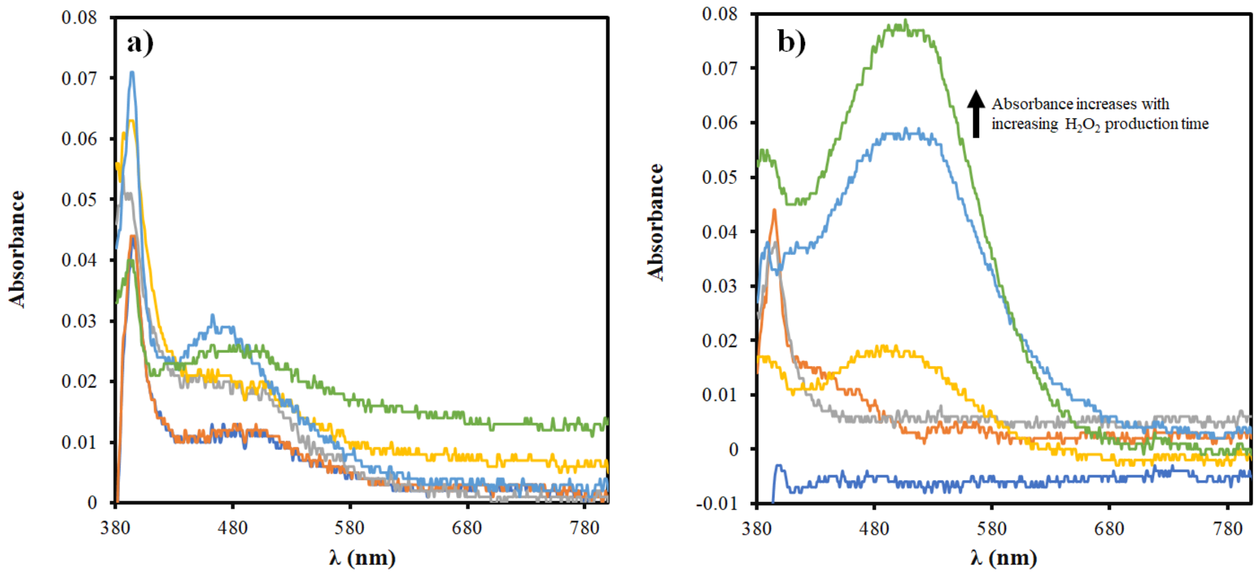

Figure S7. Absorption spectra for the colorimetric detection of H_2O_2 produced from **a)** PANI@CNT/CNC@textile; and **b)** AQ@PANI@CNT/CNC@textile patches.

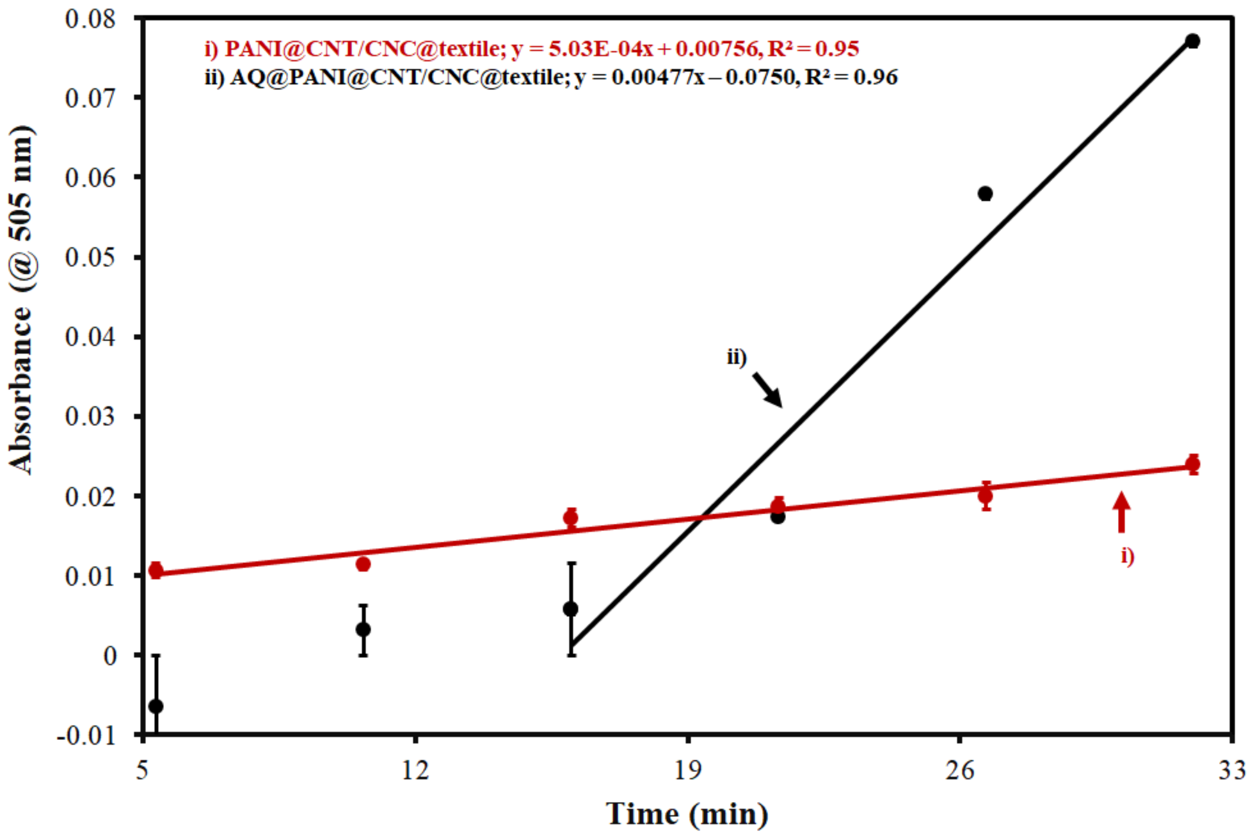

Figure S8. Absorbance (@ 505 nm) vs. electrochemical H_2O_2 generation time plot and resulting linear equations for **i)** PANI@CNT/CNC@textile; and **ii)** AQ@PANI@CNT/CNC@textile patches.

**Time (min)**	**CNT/CNC@textile**	**PANI@CNT/CNC@ textile**	**AQ@PANI@CNT/CNC@ textile**
**0**	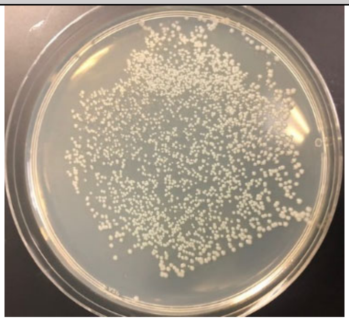	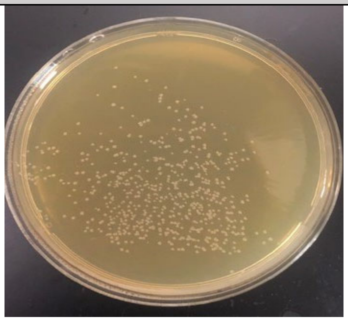	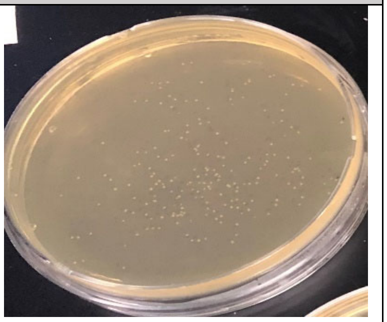

**5**	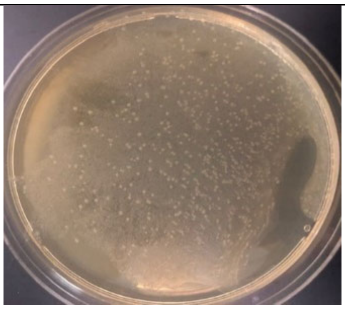	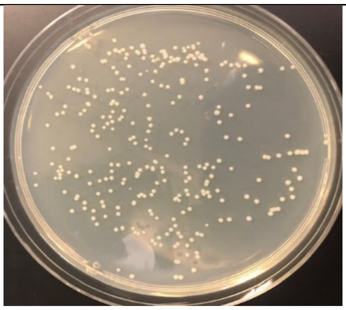	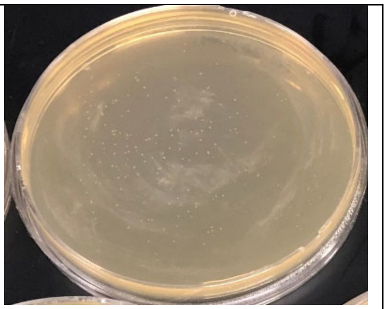
**11**	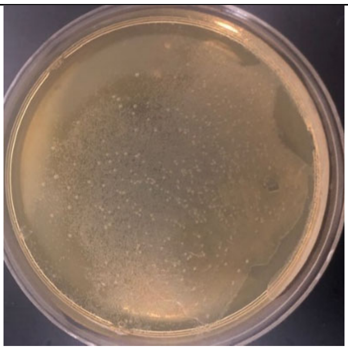	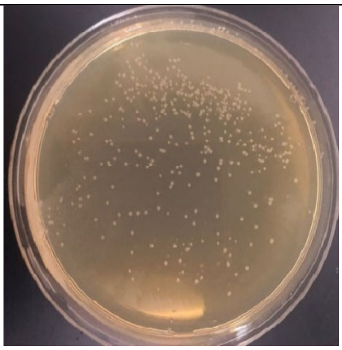	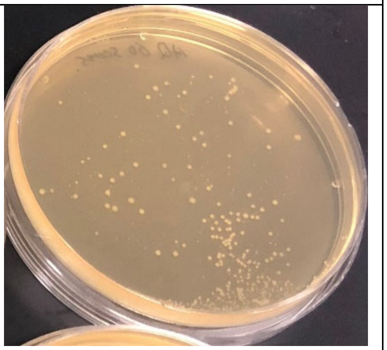
**16**	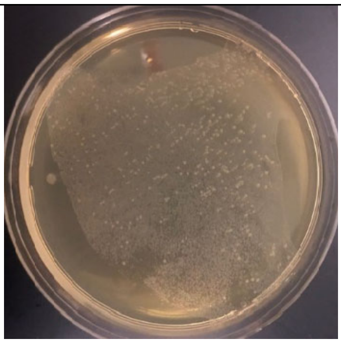	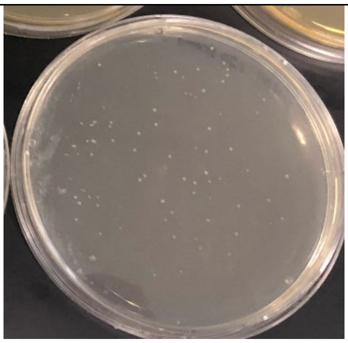	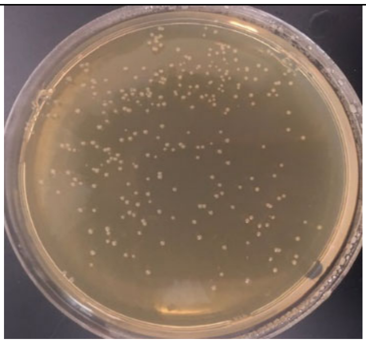
**21**	**N/A**	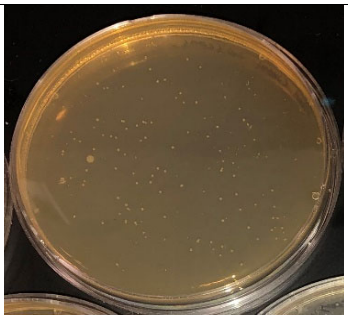	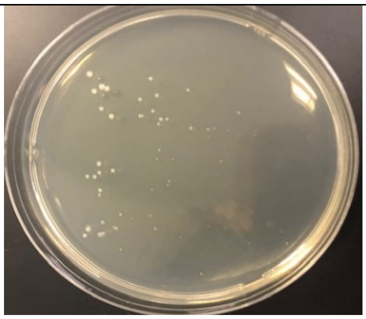
**27**	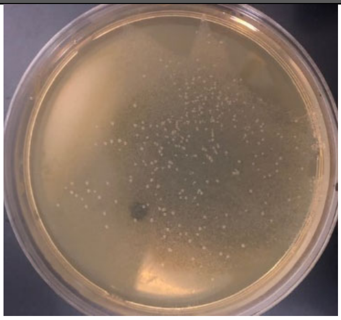	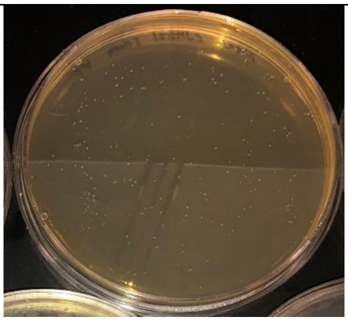	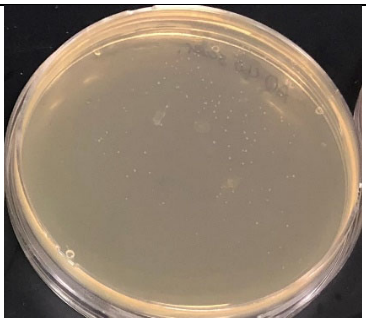

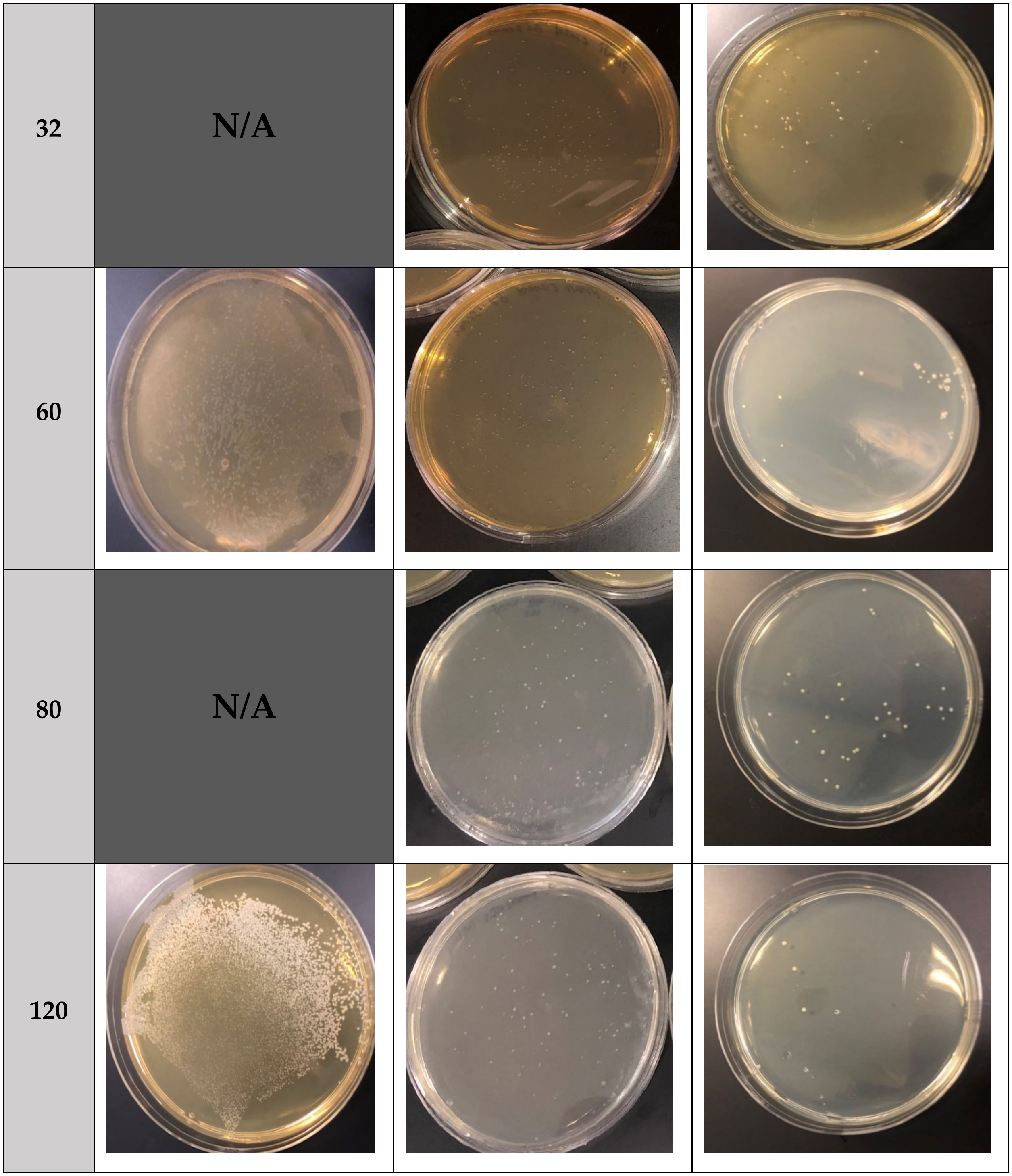

Figure S9. Camera images of *E. coli* colonies taken following various lengths of exposure to H_2O_2 production from CNT/CNC@textile, PANI@CNT/CNC@textile, and AQ@PANI@CNT/CNC@textile patches.
